# Extracts and Gleanings

**Published:** 1872-05

**Authors:** 


### PART II.

### Abridged Extracts and Gleanings from our Exchanges

*MULTUM IN PARVO.*

SPINAL CUPPING IN TEDIOUS LABOR.—The beneficial effects of spinal cupping in protracted and tedious labor is of no modern suggestion. We well remember seeing it highly recommended some twenty-five years ago, and, on being favorably impressed with the idea at the time, immediately adopted the practice, and have continued it uninteruptedly ever since, and that, too, with increased confidence. We have resorted to this remedial measure in hundreds of cases, and rarely ever at the cost of disappointment; but on the contrary, with prompt and signal success. Why the practice has not incurred general favor with the profession is owing, we think, to inefficiency of the operation in at least two particulars. Many, doubtless, on first trial, used only the medium sized ordinary cupping glass, and finding the effects nugatory, abandoned the measure. Now, we have long since learned that a single glass tumbler or large goblet, well applied just above the sacral region, is worth half a dozen common cupping glasses. Then, we say, use a well exhausted cup, or rather tumbler, as large as space will admit, and rest assured a failure in any given number of cases will be only exceptional. Again, much depends upon the length of time the glass is allowed to remain attached; 5 or 10, or even 15 minutes duration, will not do, but persist in keeping it there at least half an hour, unless, as is often the case, the desired end is sooner attained. In a case where a rigid, unyielding os uteri is the prominent obstacle, apply the glass, and after fifteen or twenty minutes' drawing, let the patient have ten or fifteen drops of gelseminum, and our word for it in forty-nine cases out of fifty, the difficulty will speedily succumb. We sometimes find, on being obstetrically summoned, that our patient has been harrassed and fatigued for hours, or even days, with "cutting" pains, which, we are gravely told by "Granny," are "doing no good." After due attention to the state of the bowels, the over cupping, with ten or fifteen grains of chloral will soon bring about relaxation, lessen the sensibility, and promptly cause a favorable progress of matters. This means will also speedily relieve spurious efforts without interfering in the least with true labor pains. In irregular or "hour glass" contractions, we invariably resort to spinal cupping and gelseminum as a preparatory auxiliary to our usual procedure in such cases.

It is granted on all hands that ergot is somewhat uncertain in action, and not unfrequently failing of its therapeutical effects. When this happens, we often find that the timely application of a glass speedily promotes the action of the parturient. In a word, we verily believe spinal cupping, when properly done, is of undoubted value in many conditions of the lying-in patient. The various indications for its use will be readily suggested by the judgment of every judicious practitioner. Our chief aim in this brief article is to insist on a more general resort to the operation by my co-laborers in the field of practice.

G. D. Hodge, M. D.

Holly Springs, Ark.

From Our Private Prescription Book.—

R.—Sulph. Ether . . . oz. i.
Camphor . . . . q. s.—Add.

The camphor almost to saturation—pour lightly over an ordinary pane of window glass, and a few moments exposure to the air will give a finely lavigated, spongy, snow-like powder. Transfer to your bottle, and keep well corked. Camphor thus treated is left slightly etheralized, and materially enhanced in medical properties. Well suited for saddle-bags use, as this does not agglutinate or form into hard lumps on agitation, as is the case with camphor pulverized by the addition of Alcohol.

R.—Iodoform, . . . . gr. i.
Iron, . . . . gr. i.
Lupulin, . . . . gr. ij.—Beat.

To pill mass, without any adjuvant—make out pill. Give one or two every night as a pain-relieving, sleep-producing and tonic alterative. An excellent pill given as an auxiliary to the iodides and bromides administered through the day.

R.—Iodoform, . . . . gr. j.
Iron, . . . . gr. j.
Sulph. Bebeerin, . . . gr. ij.—Make pill.

S.—One pill to be taken morning and noon, after meals, and three at bed time. The above pill, conjoined with other appropriate treatment, we have found very efficient in almost all chronic uterine ailments.

| | | |
|---|---|---|
| ℞.—Yolk of egg (by weight), | . . . | 4 parts. |
| Glycerine, " | . . . . . | 5 " |
| Iodoform, | . . . . . | gr. 40. |

First rub up the iodoform with the egg, and then the glycerine. Shake well, and paint the affected surface with a soft brush or feathers as often as judgment may dictate. On drying, an impervious film or layer is formed, which may be increased by repeated applications. We most confidently recommend the above as an admirable local remedy in chronic ulcers or sores (whether cancerous or otherwise), painful cutaneous diseases, and especially so in erysipelas. In most instances a drachm or two of collodion may be advantageously added to an ounce of the above. A few grains of tannin, or even a thick mucilage, in lieu of the iodoform, furnishes a harmless though sovereign remedy for sore nipples. Again, one part of carbolic acid to six of the glycerine compound makes a truly efficient application for scalds and burns. Lastly, the glycerine and yolk, in the above proportion (as recently published in the Philadelphia Journal of pharmacy), serves as a good vehicle for the internal use of chloroform in congestions, colic, etc., and of appropriate astringents or anodynes in typhoid fever, diarrhœa and dysentery. Try it, will you, and report through THE COMPANION. J. KNOX HODGE, M. D.

Holly Springs, Ark.

BROMIDE OF POTASSIUM IN EPILEPSY.—Believing the bromide of potassium to be entitled to the appreciation of a specific in epilepsy, if there is a specific for any disease, I will give you the result of its employment in the rather remarkable cases which have fallen under my care, with the view of attracting the attention of the profession to its use in the above named disease.

Case 1.—L. M., a stoutly built youth of about fifteen years of age, came under my treatment about the 1st of March, '72. Had a fit on him at the time. Was told by his mother that he had been afflicted with them since the age of one or two years, and the fits had become more frequent as he grew older. She had never been able to confine him to study, or to do any work. After administering to him medicines directed to the general system, I put him on the bromide of potassium, 15 grains administered thrice daily, alternating with hydrate of chloral in 10 grain doses at night. The treatment has not been changed, and, though the fits were frequent up to the time of his commencing the treatment, he has not had an attack since, and is now able to do any kind of outdoor work without interfering with his general health.

Case 2.—A young girl, Miss G. G., about fifteen years of age, fell under my care about two weeks later than the preceding case, being very weak, pale, and anæmic; and was told by her parents that she had been suffering with epilepsy since infancy, and that, although they had been told that upon the appearance of menstruation, her fits would cease, that they had continued to grow more and more frequent, though she had been menstruating for twelve months or more. Believing the bromide to be the most efficient remedy, I determined to give it a fair trial in her case *per se*. Directing 20 grains to be given thrice daily, with no other treatment—and, although she had had an unusually long and severe fit the day before, lasting upwards of two hours, and had averaged one or more a week for a loug time past—she has never had a return of the attacks since she commenced the treatment; and whereas, her parents were afraid to allow her to go away from home by herself, for fear of an attack, she now visits her neighbors alone, and can walk a mile or more with ease. Her health in every respect is very much improved, and I attribute her rapid improvement solely to the use of the bromide of potassium.

Respectfully, J. S. Wharton, M. D.

Lynchburg, Va., May 15, '72.

Clinical Treatment of Pneumonia.—Professor Lebert, of Breslau (*Transln. in the Clinic*), says that the ground basis of the treatment of pneumonia, so long as its peculiar manifestations exist in moderate grade, threatening no immediate danger, should be eminently expectant, chiefly only dietetic-hygienic. He has treated more than forty cases during the past year, for the most part negatively, and finds that patients feel subjectively much better, by this method, than under the former plans with antimony, digitalis, veratrine, etc. They are spared, too, in this way, the temporary disease caused by the remedy itself. Warm bed-covers are condemned. In Breslau it is the custom to sleep between feather-beds. These have been thrust out in his clinic, and patients become accustomed to the frequent opening of the windows by day, even in the coldest weather of winter. Notwithstanding the lessened appetite, the patient, even in still increasing fever, is not to be allowed to fast entirely, which is too generally the rule; light gruels of barley and oat-meal, in cases of violent thirst in greater quantities, with nourishing and alleviating drinks. In intense fever, hard pulse, if no diarrhœa be present, acid drinks are more agreeable; the best is cold lemonade, only moderately sweetened and prepared from fresh lemons. Mixtures of fruit syrups, raspberry juice, etc., are occasionally temporarily agreeable, but are usually not long palatable. Many patients prefer warm drinks, and they suit better where there is inclination to racking cough or diarrhœa. Common tea, from a teaspoonful to three-quarters of a cup, is a good drink in relieving irritation. Seltzer-water, with milk, is refreshing when the fever begins to diminish, and blood no longer presents in the sputa. When the appetite is very poor, infusions of quassia and decoctions of Iceland moss are of value. In very reduced patients, in typhoid conditions of the disease, he generally adds to the teas or other drinks a teaspoonful or two of either syrup to the glass, or desertspoonful of Rhine, red or Hungarian wine. Under such conditions the combination of wine and lemonade is excellent.—*N. Y. Medical Record.*

Nitrate of Amyl in Colic and Enteralgia.—Dr. T. Jones (Practitioner) says: "In these affections such symptoms as the cold skin, pale face, slow pulse, and spasmodic pains of an ordinary attack, indicate the employment of amyl. More especially would it be likely to be useful in those severe attacks which are accompanied with clammy perspiration, dusky hue, and the general signs of collapse. Dr. Anstie last year stated that he had used it in two or three cases of spasmodic cramps associated with flatulence; and he is reported as having said: 'There can be little doubt in my mind that amyl is a prompt relaxer of spasm in the alimentary canal.'

"Spasm of pharynx or œsophagus, biliary and renal colic, vesical and urethral spasm, may perhaps yield to the action of amyl. Hiccough, too, judging from its spasmodic nature, may also be expected to be checked by the remedy."

Carbolic Acid Inhalations in Phthisis.—Dr. James M. Keniston (*Boston Medical and Surgical Journal, March* 28*th*,'72) in speaking of a case of phthisis, says: Dr. Allen advised the inhalation of the following mixture three times a day:

| | |
|---|---|
| Carbolic acid crystals - - - - | dr.iv. |
| Aqua - - - - - - - | oz.ix. |

This was continued during the patient's life, and gave him so much comfort that he would have given up anything sooner than his inhalation.

For a few days after he began, the amount of matter expectorated increased, but after that, diminished. The cough also became less painful. He was told to drink all the milk he could, and eat plenty of butter, and anything else he felt an inclination for. He took no whisky, but occasionally a little wine. The inhalation also seemed to improve his appetite, formerly very poor.

Two troublesome symptoms remained—the night-sweats and sleeplessness. The latter was due partly to the former, and partly to the fits of coughing. The mineral acids, astringent baths, and, in fact, all the usual remedies were tried, but with no effect. I finally resolved to abandon any attempt to relieve the sweats, and only try to give him a good night's rest. On December 14, I ordered 15 grs. of chloral at bed-time, and gradually increased it to 20 grs. To my surprise, the night-sweats ceased at once, and never appeared again during his life, which lasted two weeks. He generally slept eight hours, occasionally awakening to cough, but always falling asleep again at once. I have no theory to offer in regard to the way in which the chloral effected this improvement, and can only state the fact.

Diagnosis of Glaucoma.—The following rules are given by Mr. Hart: The ophthalmoscopic test affords the crucial evidence of the disease, but it need not remain unsuspected for want of a few simple rules. 1. Wherever there is spontaneous dilation of the pupil (generally ovoid), hardness of the eyeball, pain, and dimunition of vision, acute glaucoma may shrewdly be suspected. 2. Wherever there is spontaneous dilation of the pupil, accompanying slight redness of the eye, with hardness of the ball and affection of the vision, subacute glaucoma may be suspected. The previous history generally includes the following subjective symptoms: rapidly increasing far-sightedness, haloes seen around lights at nights, and occasional pains in the eyeball. I leave out of view any opthalmoscopic or other of the optical tests of circumscription of the field of vision, for experts will already be on their guard. The external characters to which I refer in these short rules have been more or less typically conjoined in all the cases which have come under my notice. I am satisfied that it will be very useful that they should, in the minds of all practitioners, be so associated with glaucoma (to which they belong) as always to suggest by their occurrence the probable nature of the disease, and to lead to decisive investigation, and, if necessary, timely action. More eyes have been, and apparently still are, lost by the neglect or misapprehension of glaucomatous affections than from any other single cause.—*Boston Medical and Surgical Journal.*

Art of Prescribing for Children.—Dr. John O. Reilly *American Practitioner, April,* 1872) says: There are two points in the general medication of children to which I wish especially to call your attention. One is the subject of thirst, the other is the intervals at which medicine should be given.

In quite a number of infantile diseases the stomach is very capricious, and to keep it quiet is one of our greatest troubles. This difficulty is often caused by the attendant not understanding the difference between hunger and thirst. The child is fretful, and cries and pulls at its mother's breast; and she, willing to do anything that soothes it, permits it to nurse. It sucks, and in a few moments rejects the milk; but cries again, and the mother again yields it the breast only to have the stomach again reject its contents, and thus the fight goes on until the infant is exhausted. The doctor gives medicine to quiet the irritable stomach, and the mother counteracts its effect by overfeeding. What I wish to express is the fact that the child is not hungry; it does not want the breast; but is thirsty and wants drink. In health the breast is food and drink, but in disease the craving is that of thirst, not of hunger, and the stomach which rejects the milk because it is unable to digest it would be calmed by a cool beverage. In other words, were water given to the child in place of the breast, the stomach would be relieved, and in many cases the child saved. The difference between thirst and hunger in the infant is a point well worth noting.

Medicine should be given to infants in small but often repeated doses. The interval should be only half as long as that for the adult. The reason for this is that the digestive organs of the infant act much more rapidly than those of an adult, and a medicine to have its effect kept up must be supplied in accordance with its entrance into and disappearance from the system.

On the Tteatment of Bedsores.—Dr. William A. Hammond, in his Treatise on Diseases of the Nervous System, says, in the chapter on "Spinal Meningitis," that for the cure of bedsores the method recommended by Dr. Brown-Sequard may be used. It consists in the alternate application of sponges, one of which is saturated with hot water and the other with cold water. This should be done for five or ten minutes every day, and the effect is to increase the activity of the circulation of the part and to promote the formation of granulations. But Dr. H. generally prefers the method by galvanism first suggested by Crussel, of St. Petersburg, and which he used in indolent ulcers with almost invariable success in 1859, when surgeon to the Baltimore Infirmary. During the last six years he has employed it to a great extent in the treatment of bedsores caused by diseases of the spinal cord, and with scarcely a failure; indeed he may say without any failure, except in two cases where deep sinuses had formed, which could not be reached by the apparatus. A thin silver plate, no thicker than a sheet of paper, is cut to the exact size and shape of the bedsore. A zinc plate of about the same size is connected with the silver plate by a fine silver or copper wire six or eight inches in length. The silver plate is then placed in immediate contact with the bedsore, and the zinc plate on some part of the skin above, a piece of chamois-skin soaked in vinegar intervening. This must be kept moist, or there is little or no action of the battery. Within a few hours the effect is perceptible, and in a day or two the cure is complete in the great majority of cases. In a few instances a longer time is required. He has frequently seen bedsores three or four inches in diameter, and half an inch deep, heal entirely over in forty-eight hours. Mr. Spencer Wells states that he has often witnessed large ulcers covered with granulations within twenty-four hours, and completely filled up and cicatrization begun in forty-eight hours. During his recent visit to this country he reiterated his opinion that it was the best of all methods for treating ulcers of indolent character and bedsores.—*Rankin's Half-Yearly Abstract.*

Treatment of Chronic Diarrhea.—Dr. Eustace Smith, in his work on Wasting Diseases of Infants and Children, recommends the following plan of treating obstinate cases of chronic diarrhea: "All food must be stopped, and the child must be nourished in the following way: A piece of raw mutton or rump steak, free from gristle or fat, is finely minced, and is pounded in a mortar till it is converted into a pulp. The pulp is then strained through a fine sieve or a piece of muslin to remove the blood-vessels and cellular tissue. Of the meat so prepared a teaspoonful is given at regular intervals four times in the day, and every day the quantity administered is gradually increased until half a pound is taken each day in divided doses. During this treatment *no other food of any kind must be allowed*, and no fluid but thin barley-water, or a drink made by mixing the unboiled whites of three eggs in a pint of water, sweetening it, and flavoring with a little orange-flower water. This diet usually causes the motions to have an intensely offensive smell; but this is of no consequenoe, and the parents should be warned of its liability to occur. The patients themselves often like this food, and take it eagerly. If, however, as may happen, they show any repugnance to it, the pulp may be sweetened with white sugar, or a little confection of roses may be added to make it more palatable, or it may be given in a small quantity of veal-broth. As medicine, we must give at the same time the bismuth and chalk mixture, with the addition of one drop of tinct. opii to each dose."—*Rankin's Half-Yearly Abstract.*

Bromide of Potassium in Sick Headache.—Dr. L. P. Yandell, Jr., says: In sixty-grain doses, repeated hourly until relief from pain ensued, we have found bromide of potassium but little less reliable in sick-headache than quinine is in ague.

Belladonna as an Antigalactic.—T. A. Reamy, M. D., of Cincinnati, reports in the *Clinic*, a remarkable experience of the power of belladonna to arrest the mammary secretion. During a period of fifteen years he has treated twenty-two cases, in twenty of which the success was perfect. In two the remedy was powerless. In one of these abscesses formed, an accident, which had occurred after a previous confinement, leading, Dr. R. thinks, to changes of structure, from which the breast had not recovered. Prof. R. attaches great importance to the *time* when the remedy is employed, to the *preparation* and *mode of application* of the drug. The remedy should be applied immediately after delivery, and continued throughout the forty to seventy hours which supervene between that time and the secretion of milk. An aqueous solution of the alcoholic extract, fifty grains to the ounce, Dr. R. thinks the best form; and strips of muslin saturated with this solution applied to the parts, and covered with oil-silk, the best mode of using the medicine. He lays great stress on making no friction of the glands, because of its tendency to excite secretion of milk. For the same reason he enjoins that the breasts shall not be emptied of colostrum, or of any milk which may have been secreted, and that pups, pumps, and nurses' mouths, should not be allowed to come near. If on the second or third day there should be fever, accompanied with pain or fullness of the mamma, as will often occur, no alarm need be felt. The symptoms will subside in a few hours.

Diptheria and Scarlet Fever Treated by Ice.—Dr. A. S. Von Mansfelde (*Medical and Surgical Reporter*) says: Last spring I was called to the house of Mr. N., of this city, and found three patients, two daughters, aged respectively 12 and 10 years, and a son about six years old, all of whom presented symptoms, more or less severe, of *diptheria.* No pleasant sight, but less troublesome by the trust in my *good will* to help. The intelligent mother was directed to make *ice cream*, procure *ice*, and feed the former and apply the latter to the necks of the sufferers

(*a la mode de* Dr. Corson) and permit them the use of ice, as their thirst should call for it. The good nurse shook her head a little, owing to a case, in the same family, four years ago, which we treated quite differently at the time, the patient surviving in spite of the doctor. She set at work, nevertheless, to do our wishes, and we went our way, feeling like a student fifteen minutes before his final examination for degree. Next morning we came, and wished Dr. Corson or Dr. Snyder had been with us. The miracle wrought by ice and ice cream was almost too wonderful. Our family thought nobody ever did the like, and we permitted them to think so. In a few days all three were perfectly relieved of the trouble, and have not suffered the slightest inconvenience since from that source.

In June, a little six-year old boy sickened with scarlet fever, and, though being attended by allopathy, homeopathy, and other "pathies," died of the disease. This being transplanted into the next house, took with it a younger child; almost all the members of both families, as far as I could ascertain, suffering from scarlet fever or sore throat, at the same time, and later. In the third house from starting point, or next house to the last named victims above, it infected a 15 year old boy, whom I had attended three years ago with typhoid fever, which he stood very bravely; but this time he soon went from bad to worse. We had read Dr. Corson's letters in regard to this matter. This, and the knowledge of some of the medicines given to the other cases, gave birth to the resolution: No MORE OF MEDICINE THAT FAILS, *and something that has not had a chance of failing as yet.* Our patient presented a most discouraging condition. When we saw him, he was in delirium, had sores on lips and gums, and exudation from the ulcerated throat, that almost prevented the respiratory process; high fever and incontinence of the excrements, and worst of all, a step-mother that cared little for him, who, with her lady friend, pronounced the patient a dead boy. With this material we set to work, with the aid of the father in enforcing our directions. We put him into a *cool* bath, which brought him quickly to his senses (by the way, we found this the best remedy in delirium of typhoid fever); then we applied ice to the neck and—*kept it there*, permitting him ice and ice water to quench his thirst. No medicines. Next morning the throat was somewhat better—this affection seeming the greatest danger to the life of the boy—but the body was weaker; in consequence gave small doses of quinine in tinct. ferri chlor., and ordered beef tea, eggs, and a little brandy. In the evening he was a little more feverish, therefore gave the bath, and again with no more medicine, than above mentioned, we had the great pleasure of seeing our patient live and grow strong in body, and in his confidence of *our treatment* (Dr. Corson's). We omitted a minute description of the disease as it was the same presented in any patient sick with a grave form of scarlatina, only laying stress upon the treatment, its being a specific for the same—ICE *or its equivalent*—cold.

TREATMENT OF GONORRHŒA.—Dr. C. Van Wyck (*Western Lancet*, 1872) in referring to cases of gonorrhœa treated by him, remarks: In each of these I adopted the abortive treatment, using a small piece of sponge attached to a wire and passing through a silver tube, which I introduced some two inches within the urethra and thrust out the sponge previously saturated with a solution of argent. nit. grs. x, to the ounce of water, with which, by a rotary movement, I freely swabbed out the canal at least two-thirds of its extent. I then ordered the following to be used as an injection after the expiration of a few hours:

| | |
|---|---|
| R.—Zinci sulph., - - - - - | scr.j. |
| Ac. carbolic, - - - - - | gtt.xx. |
| Morph. sulph., - - - - | grs.iij. |
| Glycerin., - - - - - | oz.j. |
| Aq. pur., - - - - - | oz.vij—M. |

Use as an injection three or four times daily.

These eleven cases, treated in this manner, are convincing proof to me that carbolic acid, in combination with astringents and anodynes, is as near being a specific in uncomplicated cases of blennorrhagia, as any remedy believed to be such in the domain of the Materia Medica.

OIL OF CADE IN ECZEMA.—Dr. Newcombe sends the following note to the *Practitioner:* "Having found the above preparation particularly efficacious as an outward application in many cases of eczema of the scalp, especially those which so closely resemble pityriasis, I was induced to try it in eczema of a dry character in other parts of the body, and did so with marked success. I have lately used it in a case of 'psoriasis diffusa,' extending from both knees down the front of the leg, and also appearing on the fore-arm, which had baffled the ordinary plan of treatment for a long time, and the improvement was so immediate that it could not be entirely due to the arsenic which the patient had been taking. I direct my patients to rub it well over all the spots every night with a camel's-hair brush."

Atomized Turpentine-Water in Chronic Bronchitis and Consumption.—Dr. S. Goodwin, of Victoria, Texas, writes us (*Medical News and Library*) that he has been lately using turpentine-water, inhaled by means of an atomizer, with signal advantage, in cases of chronic bronchitis and consumption, where there was copious expectoration of either mucus or pus, with hemorrhage and violent cough. The turpentine-water is prepared from spirits of turpentine by magnesia in the same way that aromatic waters are commonly prepared by druggists.

Carbuncle Treated by Cups.—M. Hamon (*Bulletin Generale;* from the *Revue Med.*, June, 1870) recommends the application of cups to carbuncles after they have been freely lanced. From two to three ounces of blood should be removed in this way. He claims that the phlegmonous affection may in this way be aborted if it is done in time, and that the sloughing and profuse suppuration, which are the consequences of the modes of treatment most in vogue, are avoided by it.—*Medical Times, Dec.* 1, 1871.

Chloral in Cholera.—M. Reichard (*L'Union Medicale;* from the *Gazette Medicale de Strasbourg*) has employed chloral in cholera to fulfil the following indications: 1. To relieve the cramps at the commencement of the disease. 2. To assuage the precordial pain at its close. 3. To arrest vomiting; and, 4. To procure sleep. He says that not only are all these results obtained, but the success of the treatment also surpassed expectation.—*Ibid.*

Braun's Colpeurynter as a Tampon.—In discussing the merits of tampons, in the Gynæcological Society of Boston, (*Gynœcological Journal, Dec.* 11), Dr. Bixby said: For his own part, excepting in cases of sudden emergency, where there was nothing else at hand, he should endeavor to use Braun's colpeurynter. Any physician who had ever used that instrument once, and appreciated with what facility it could be introduced, filled with ice-cold water, and emptied by merely turning a valve, all without the slightest discomfort to the patient, would never again torment a poor woman, whose parts were swollen, inflamed, and not unfrequently excoriated by previous manipulation, by using rags.

Inunction in the Treatment of Disease.—The Italian physicians appear (*Journal fur Kinderkrankheiten*, Heft 3 und 4) to be very fond of the use of olive-oil by inunction in the treatment of the diseases of children in which the functions of the skin are depressed. The whole body is rubbed with tepid olive-oil every twelve, six, or four hours, according to the urgency of the case. The child is afterward wrapped in flannel. The *Gazzetta Medica Ital. Provinc. Venet.* asserts that these inunctions are much more efficacious than the warm bath, because—1. They restore more completely and permanently the fu ctions of the skin. 2. The disadvantage of a reaction is avoided by their use, and the layer of oil protects the surface of the body from direct contact with the atmospheric air. 3. The oil prevents organic combustion, and in this way aids nutrition. 4. The inunction with oil does not produce a depressing, but, on the contrary, an exhilirating effect. In some cases an improvement in the symptoms has been noticed twenty minutes after the operation, but generally not until forty-eight to seventy hours.—*Medical Times, Dec.* 11, '71.

DIPTHERITIC ENTERITIS (?).—In the proceedings of the Gynæcological Society, Boston (*Gynæcological Journal, Dec.* 1871), Dr. Martin mentioned the case of a woman suffering from some unusual disease of the rectum, who brought with her a bottle containing a quantity of peculiarly colored membranes resembling egg-shells, some of them as large as a fifty-cent piece, which she had been passing for two years. He had not yet examined the case very thoroughly, but had ordered weak injections of nitrate of silver. He intended in due time to rupture the sphincter and examine the case more thoroughly.

Dr. Bixby stated that he had had under his care a patient suffering probably from the same trouble. The patient, a woman aged forty, married, had never had any children, had been sick for twelve years, and for two years had been passing this peculiar membrane, or cast, resembling that spoken of in Dr. Martin's case. Upon referring to different authorities, among others Drs. Simpson and Watson, he was disposed to class it under the head of eruptive diseases of the intestine. In his patient the disease was localized and seemed to occur in paroxysms; during these, the seat of the trouble was very sensitive upon pressure, and not unfrequently caused severe tenesmus and was attended with constitutional disturbance, such as fainting, depression, and vomiting, from which she did not rally for weeks afterwards. Generally, about one week after the occurrence of the paroxysm, there passed large quantities of this membrane, which Dr. Watson describes as resembling chewed walnuts, sometimes in the form of long shreds, and in others in that of round balls which could be unravelled, and were found to be composed of these different-colored shreds. He had therefore treated this case with Fowler's

Solution internally, and counter-irritation externally along the tract of the intestine, kept up for some time; also by soothing injections, such as glycerine, chlorate of potash, gum, or starch water, before and after the stool. In the course of a few weeks there had been a decided change in the symptoms, and when last seen, the patient was not passing any more membranes, and was better in many other respects. She had always complained of obstinate constipation. The case was a very complicated one, the patient having suffered from uterine and other troubles.

Glycerin in Putrid Sore-Throat.—I have found this an invaluable remedy in putrid sore-throat, as well as in many other affections. Not long since a case occurred in which its healing properties were fully tested. The patient, a little girl, seven years of age, had been suffering several days before I saw her, and the various remedies employed had made no impression on the disease. As it was with great difficulty and pain she swallowed, and her pulse being very weak and quick, it was important that the remedy adopted should possess healing, nourishing, and antiseptic properties; and glycerin, possessing these properties, was administered in teaspoonful doses every six hours. The first dose caused some smarting, the second less, and before giving the third there was obvious improvement. The case was dismissed in three days.—*Dr. J. D. Palmer, in Amer. Jour of Pharmacy.*

Carlo Paresi's Method of Concealing the Taste of Cod-Liver Oil.—Cod-liver treated in the following manner becomes amber-colored, and has the odor and taste of coffee. The fishy taste is almost entirely masked.

Take 400 parts of cod-liver,
10 " animal charcoal,
20 " roasted coffee (ground).

Mix them thoroughly in a glass matrass, and heat in a water-bath for quarter of an hour, to temperature of 50 to 60 deg. C, taking care to stop the mouth of the matrass. Then take the mixture off the fire and allow it to stand for three days, shaking it occasionally. Then filter, and the oil is ready for use. It must be kept in well-stopped bottles.—*Bulletin General de Therap, Nov.* 1871.

Death from Syrup of Poppies.—In the London *Pharmaceutical Journal* of October 3d are detailed two cases in which death was caused by syrup of poppies. In one case a teaspoonful was given to a child eighteen weeks old, at 6 P. M., death occurring about 8 A. M. the next day. The other victim was a child five weeks old, to whom three parts of a teaspoonful were given.

Veratrum Viride in Inflammatory Affections. By J. Lewis Smith, M. D., Curator to the Nursery and Child's Hospital, New York. In the treatment of inflammatory affections, Dr. Smith recommends the use of aconite or veratrum viride as a substitute for blood-letting. The following paragraph refers to lobar pneumonia:

If the previous health of the patient has been good, his age above three years, the attack primary, and the inflammation is, in part at least, in the first stage, aconite or veratrum viride, properly employed, is serviceable. Either one is an efficient substitute for blood-letting. Some prefer aconite as less depressing than veratrum, and it is known to be a favorite remedy of homœopathists. I have ordinarily employed the veratrum, prescribing the tincture in doses of one drop every three hours to a child of five years. It can be given dropped in sweetened water, or in syrup of tolu. Its effects should be carefully watched, and it should be omitted, or given less frequently, when the pulse is reduced to near the natural frequency.—*Treatise on the Diseases of Infancy and Childhood.*

Phosphorized Ether in Depression.—In a translated article by Dr. Chavett (*Chicago Medical Times*), several illustrative cases are given of the stimulative effects of phosphorus in an emulsion with ether, from which we extract the following:

Emanuel Jobert, æt thirteen, was attacked with typhoid fever. Symptoms, January 20th, were as follows: Loss of voice and consciousness, tremor of the tongue and inability to protrude it beyond the lips, deglutition extremely difficult, eyes closed and sight gone, respirations very labored, pulse feeble, unequal, intermitting, trembling, and variable, excessive involuntary colored dejections of a dark, sanguinolent character, great difficulty in producing rubefaction of the dermis, coldness of the extremities, gangrene of the parts upon which the decubitus exists ; in a word, he seemed about expiring. His morbid condition was not modified by rubefacients, astringent enemata, tonics, frictions and diffusible stimulants. January 25th, Dr. Depaule prescribed the following:

| | |
|---|---|
| R.—Phosphorus, . . . . . . | grs.ij. |
| Ether, Sulph., . . . . . . | drs.i. |
| Emulsio. Amygdal. Dulc., . . . . | oz.vj.—M. |

Sig. Semi cochl. omni hora. (Size of spoon not mentioned.)

Much difficulty was experienced in causing the patient to swallow the first four doses, so difficult was deglutition ; the successive doses were swallowed with less trouble. The patient soon began to utter plaintive cries, vivacity returned to the eyes, which had been closed for eight or nine days, and a great heat and general diaphoresis, with continual agitation, were manifested, with a severe irritation of the œsophagus, stomach and intestines. The phosphorated emulsion of which the patient had taken about one-fourth, was discontinued. On the 27th, a notable amelioration was apparent, and restoration to health followed progressively, notwithstanding numerous abscesses in various parts of the body, and the enormous discharges resulting from the gangrenous eschars. February 22d, young Jobert was in full convalescence. *Medical Cosmos, Jan.*, '72.

Connection of Various Diseases—Variola.—The Vienna correspondent of the *Lancet* states (Medical News) "that Professor Hebra recently made two most interesting and practical remarks on the connection of various diseases. Firstly, he stated that about ninety per cent. of the parents of pruriginous children die of phthisis; and secondly, it is very common for women who have long suffered from eczema of the head to become the subjects of cancer later in life. A considerable number of cases of small-pox are now under treatment, two of which seem likely to end fatally. The Professor believes in no treatment beyond good food and air, and considers medicine even injurious, as irritating the pustules developed on the mucous membrane of the mouth. This is in marked contrast to the views of Dr. Monti, of the children's Hospital, who has recently been treating very successfully all his small-pox cases with small internal doses of carbolic acid. I may add that Professor Hebra stoutly denies any essential difference between the contagion of variola and varicella, having seen formidable outbreaks of the graver malady produced by the introduction of a solitary example of the lighter and so-called distinct disease."—*Medical Cosmos, Jan.*, '72.

Chloral in the Opium Habit.—Dr. J. E. Bowers, of St. Peter, Minnesota, writes (Northwestern Medical and Surgical Journal): "In the great majority of the cases where I have tried chloral hydrate, the remedy has fully sustained its reputation, and has done all that its friends have claimed for it. Where it has proved unsatisfactory, the failure has not been due to its want of sleep-producing power. The case, however, in which the salt has proved the most satisfactory was one of opium habit. This patient, a woman, after taking opium for fifteen years, had become completely enslaved, and was suffering all the evil consequences of the habit—as mental illusions and hallucinations of sight and hearing. She dreaded the approach of night, on account of the restlessness, wakefulness and suffering that always came with it. When she succeded in getting to sleep she was tortured with the most horrible dreams. She also had a persistent diarrhœa, and her skin was bathed with cold perspiration, always worst at night. The first night she took chloral grs. xx, and tr. hyos. dr. j, the dose being repeated in two hours —very little sleep. The second evening she took an equal quantity in one dose with much better effect. By her own request, the quantity was gradually increased till she took chloral hydrate dr. j, tr. hyos. drs. ij. The dose was continued nightly for a fortnight with a good effect, and was then gradually diminished. At present she sleeps well on grs. xxx, and has pretty well recovered from her mental disturbance."

Post-Partum Dietetic Treatment.—In the discussion of post-partum dietetic treatment, in the Obstetrical Society of Edinburgh (American Journal of Obstetrics, November, '71), Dr. Bell said that for thirty years he had followed one plan. Immediately after delivery, he gave a glass of whisky, brandy, or wine. In one case in which this had not been given, he had cause to regret the omission. He agreed in the propriety of keeping up the system, but much must of course depend upon the habits and constitution of the patient. In most cases he was in the habit of allowing butcher-meat and wine on the third day. He referred to the case of the late Princess Charlotte, who sank from exhaustion consequent upon hemorrhage. She had only water-gruel—no stimulants. In Shetland he understood that a bottle of whisky was given to the patient immediately after delivery, which she was expected to drink in the course of her recovery.

Dr. Sidey said that the state of the tongue, rather than the length of the labor, guided him in his dietetic treatment. The tongue was often dirty, even when the labor was easy. When the tongue is clean, diet of the kind recommended by Dr. Cairns might be safely given, but the same would not do for the other class.

Dr. Ritchie agreed theoretically with Dr. Cairn's recommendation (generous diet, etc.), but found that practically he frequently required to give a less nourishing diet, especially in those cases in which the tongue was foul.

Chronic Otitis.—Dr. W. L. Lipscomb, Columbus, Miss., (*Medical and Surgical Reporter*) says his favorite prescription is:

R.—Argent nit. fused, . . . . . gr.xl.
Aquæ, . . . . . . . . f.oz.j.—M.

Sig.—Apply twice a week with c. h. pencil.

He reported several cases thus treated with perfect success.

Nitrate of Amyl in Asthma.—Dr. Talford Jones (Practitioner), in writing of this anæsthetic, gives the following case of spasmodic asthma: "At twelve o'clock on the night of October 23, 1870, a woman begged that I would instantly go and see her daughter who, she said, "was in a dying state." On entering her bed-room, I saw the patient, a young married woman, half undressed, sitting on the corner of the bed and holding on to the bed-post. There was a dusky, leaden hue about her face, neck chest and hands, and a cold, damp sweat clung to her. Her body generally was cold, but her feet and legs were of an icy coldness. Her pulse could scarcely be felt. She was making violent efforts to breathe, and each inspiration was attended with marked recession of the supra-clavicular, and the intercostal spaces. Loud *rales* with sonorous rhoncus could be heard over the greater part of the chest. She tried to speak, but could only make faint gasps. The thought instantly occurred to me that the nitrate of amyl, which I had procured only a short time before, might be of use. I ran back to my house, which was close by, and returned with the bottle. Five drops of amyl were applied, on a piece of lint, to her nostrils. In half a minute her face began to redden, and in less than a minute it was deeply flushed; her heart palpitated, her carotids throbbed, warmth of body quickly returned, and her breathing became easy. The effect was marvellous, and I felt nearly as much astonished as the patient and her mother. She now became able to converse, and told me she had been subject to asthma for many years, that her father also was asthmatical, and that she had never before had such a severe attack as this. She accounted for it thus: In the early part of the day she was as well as usual; but that evening she remained out for some time in the wet, and returned home feeling damp, cold and chilly.

"In about ten minutes after the inhalation, the breathing became a little asthmatical, so we reapplied the amyl, and again she became perfectly easy, and went to bed. Next morning she told me that she had had a most comfortable night, the asthma was quite gone, and she was attending to her household duties.

About five months after this attack, March 26, 1871, I was again summoned to her. This time it was nothing more than an ordinarily severe bout of asthma, and wanting the signs of severe collapse that accompanied the first. A repetition of the amyl treatment was followed by results as speedy and effectual as in the first instance."

